# Subspecies Taxonomy and Inter-Population Divergences of the Critically Endangered Yellow-Breasted Bunting: Evidence from Song Variations

**DOI:** 10.3390/ani12172292

**Published:** 2022-09-04

**Authors:** Wenshuang Bao, Atul Kathait, Xiang Li, Kiyoaki Ozaki, Yukihiro Hanada, Alexander Thomas, Geoffrey John Carey, Jun Gou, Batmunkh Davaasuren, Makoto Hasebe, Paul Ian Holt, Lukas Pelikan, Zhongyong Fan, Siyu Wang, Xiaoying Xing

**Affiliations:** 1 College of Wildlife and Protected Area, Northeast Forestry University, Harbin 150040, China; 2 Northeast Asia Biodiversity Research Center, Harbin 150040, China; 3 School of Biosciences, Apeejay Stya University, Gurgaon 122103, India; 4 Yamashina Institute for Ornithology, Abiko 270-1166, Japan; 5 Independent Researcher, Kitami 090-0058, Japan; 6 Amur Bird Project, 04275 Leipzig, Germany; 7 Independent Researcher, Bali 80582, Indonesia; 8 Xinjiang BD Nature Co., Ltd., Urumqi 830000, China; 9 Wildlife Science and Conservation Center of Mongolia, Ulaanbaatar 14210, Mongolia; 10 NPO Sarobetsu Eco Network, Toyotomi 098-4100, Japan; 11 Independent Researcher, Clitheroe BB7 1PT, UK; 12 Faculty of Biology and Psychology, Georg-August-University, 37073 Göttingen, Germany; 13 Zhejiang Museum of Natural History, Zhejiang Biodiversity Institute, Hangzhou 310012, China

**Keywords:** Yellow-breasted Bunting, subspecies taxonomy, bird song, population divergence, *insulana*

## Abstract

**Simple Summary:**

Identifying the taxonomic status of subspecies or population with independent evolutionary tendencies is important for the targeted conservation of endangered species. Two subspecies of the critically endangered, Yellow-breasted Bunting *Emberiza aureola* have long been classified: *E. a. aureola* and *E. a. ornata*. However, populations distributed in Hokkaido, Japan, are sometimes considered another subspecies of *E. a. insulana*. By comparing vocal divergences, we found that the song of the Yellow-breasted Bunting *Emberiza aureola* has subspecies-specific properties and that *insulana* can be classified acoustically as a subspecies. Morphological and genetic differences should be tested further to confirm its subspecies status. Our results not only confirm the subspecies but also provide key evidence for targeted taxon conservation efforts for this critically endangered bird species, given that several Japanese populations have disappeared.

**Abstract:**

The critically endangered Yellow-breasted Bunting has undergone population collapse globally because of illegal hunting and habitat deterioration. It was listed as critically endangered (CR) by the International Union for Conservation of Nature (IUCN) in 2017 and designated a Class I (highest level) national conservation bird species in China in 2021. Birdsong in the breeding season is the main communicative signal under sexual selection, and song variations have long been considered critical evidence of divergence among subspecies or populations. We compared the songs of 89 males from 18 populations to test subspecies taxonomy. We found that songs of the Yellow-breasted Bunting *Emberiza aureola* are subspecies specific and that three subspecies can be clearly discriminated by song divergences. Moreover, an analysis of multiple vocal traits supports the claim that *insulana* is distinct from *aureola* and *ornata*. Finally, at the geographic population level, populations can be clearly classified in accordance with the three subspecies, although the *aureola* population in Xinjiang, China is differentiated from other populations of the same subspecies. The results of this study demonstrate that all populations and subspecies are unique and should be protected to maintain intraspecies song diversity. In addition, several specific populations, such as *insulana* populations in Japan and the Xinjiang, China population of *aureola*, need to be paid special attention to prevent the extinction of unique or local taxa.

## 1. Introduction

Identifying the taxonomic status of subspecies population with independent evolutionary tendencies is important for the conservation of endangered species because only if subspecies or unique populations can be identified in a timely manner can helpful taxa-targeted conservation measures be taken to avoid loss or extinction [1,2]. The Yellow-breasted Bunting *Emberiza aureola* (hereafter, YBB) has undergone severe population decline because of illegal hunting and habitat deterioration [3,4]. It was listed as critically endangered by IUCN in 2017 and designated a Class I conservation bird species by the Chinese government in 2021 [5,6,7].

Two subspecies of YBB have long been recognized based on morphology and distribution: *E. a. aureola* and *E. a. ornata*. *E. a. aureola* breeds in east Finland and west Russia easterly to Kamchatka; southerly to north Ukraine, north Kazakhstan, and west and central Mongolia and winters in south and South East Asia. *E. a. ornata* breeds from east Transbaikalia and north east Mongolia easterly to north east China (Heilongjiang), the Sea of Okhotsk coast, Sakhalin Is., and north Japan and Kuril Is. and winters in south China. The subspecies *ornata* is much like *aureola* but is darker with more extensive black on the forecrown [8]. Nevertheless, populations of *ornata* distributed in Hokkaido, Japan are sometimes taken as another subspecies *E. a. insulana* [9]. However, the divergence between *insulana* and *aureola* or *ornata* has never been tested. It is necessary to investigate differences among different subspecies or populations to determine whether *insulana* is an independent subspecies.

The general concept of a species is that it is a singly evolving metapopulation lineages (or more properly, segments thereof), and one of the operational criteria for assessing lineage separation is isolation [10]. A long period of geographical isolation and reproductive isolation is likely to lead to the generation of local groups (or subspecies) and differentiation, as well as the formation of new species. In most animals, behavioral traits are the most important isolating mechanism, and new adaptations are often initiated as a result of behavioral changes. As an important reference for avian taxonomy, the geographic variation in song structure is often consistent with morphological changes and affects the population genetic structure in a way that hinders information transmission and gene exchange between the populations [11]. Birdsong in the breeding season is used to attract a mate and defend territory, and song divergences among different subspecies or populations can lead to reproductive isolation and speciation in many bird species [12,13]. Therefore, along with morphological and genetic evidence, birdsong divergences are commonly used as an essential basis of taxonomy [14,15]. Because birdsong evolves under both natural and sexual selection, it is commonly considered to have species- [16,17], subspecies- [18], and population-specific [19,20] signals resulting from morphological divergence [21,22], adaptation to local acoustic environments [23,24], or stochastic processes, such as cultural drift [25,26]. Many cryptic new bird species, subspecies, and taxa have been proposed and tested according to their song variations, combined with genetic and morphological traits [27,28,29], and acoustic divergences are considered essential factors in investigations of taxonomic status or for resolving phylogenetic issues in ornithology [30,31].

Acoustic signals and cultural diversity have recently been put forward as potentially critical factors that affect conservation practices, such as successful translocation of endangered bird species because individuals from different populations may not recognize each other’s song or breed with each other [1,2]. These phenomena indicate that song divergences could be premating barriers that result in assortative mating, which promotes the formation of new taxa [32,33]. In this study, we analyzed quantitative song variations among *aureola*, *ornata*, and *insulana* at both the subspecies and population levels to investigate the following questions: (1) Does YBB song have subspecies-specific characteristics? (2) Are songs of Hokkaido populations (*insulana*) distinct from those of *aureola* and *ornata*? (3) What are the vocal differences among the different populations, and do any populations have unique songs that are worthy of attention in future conservation efforts? This research provides an acoustic viewpoint on taxonomy for special protection of targeted taxa.

## 2. Materials and Methods

### 2.1. Data Source

All recordings of YBB were collected through field recording. A TASCAM DA-P1 recorder (Tascam, Tokyo, Japan) and Sennheiser MKH 416 directional microphone (Sennheiser Electronic, Wedemark, Germany) with a 44.1 kHz sample rate and 24-bit depth were used to record songs. In the field, we recorded birds that were sufficiently widely spaced to be considered different individuals at each site; each site was visited only once to avoid recording the same individual more than once. To obtain a representative sample of each individual’s repertoire, we recorded as long as possible, until the individual either stopped singing or flew away.

### 2.2. Song Analysis

**Figure 1:**
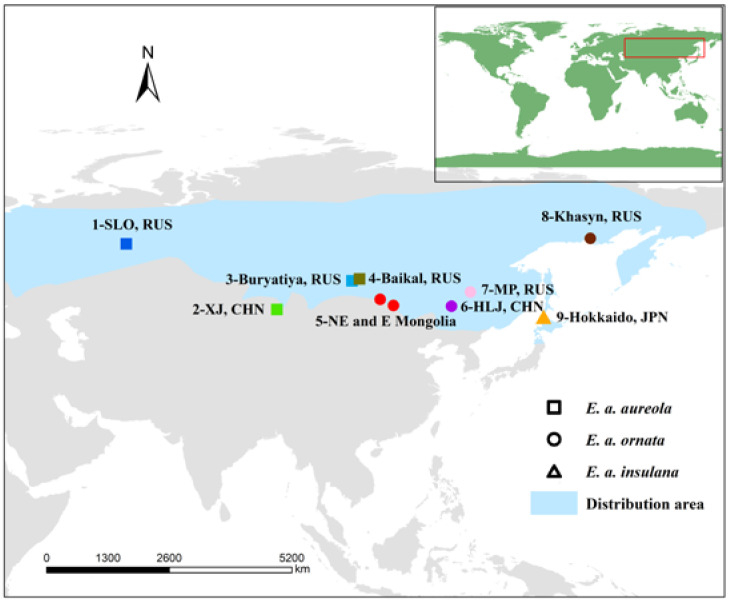
Distribution of *Emberiza aureola* taxon recordings from nine populations used in the subspecies vocal divergence analysis, with sampling sites indicated by different symbols. Squares represent *E. a. aureola* (population 1 = Sverdlovsk Oblast, Russia; 2 = Qinghe County, Xinjiang Province, China; 3 = Buryatiya, Russia; 4 = Lake Baikal, Russia), circles represent *E. a. ornata* (population 5 = northeastern and eastern Mongolia; 6 = Zhalong, Heilongjiang Province, China; 7 = Muraviovka Park, Russian Far East; 8 = Khasyn, Magadanskaya Oblast, Russia), and the triangle represents *E. a. insulana* (population 9 = Toyotomi, Hokkaido, Japan). Different colors and numbers represent different populations marked in the legend.

Recordings of different subspecies in different regions were screened by group, and recordings that contained at least one complete song were selected for analysis. After screening, we collected recordings of a total of 89 individuals from 18 geographic populations of three subspecies (Table S1). At the subspecies level, we analyzed the songs of 89 individuals from 18 populations. At the geographic population level, because we removed populations that contained only one individual, we analyzed the songs of 80 individuals from nine populations (Figure 1 and Table S1).

**Figure 2:**
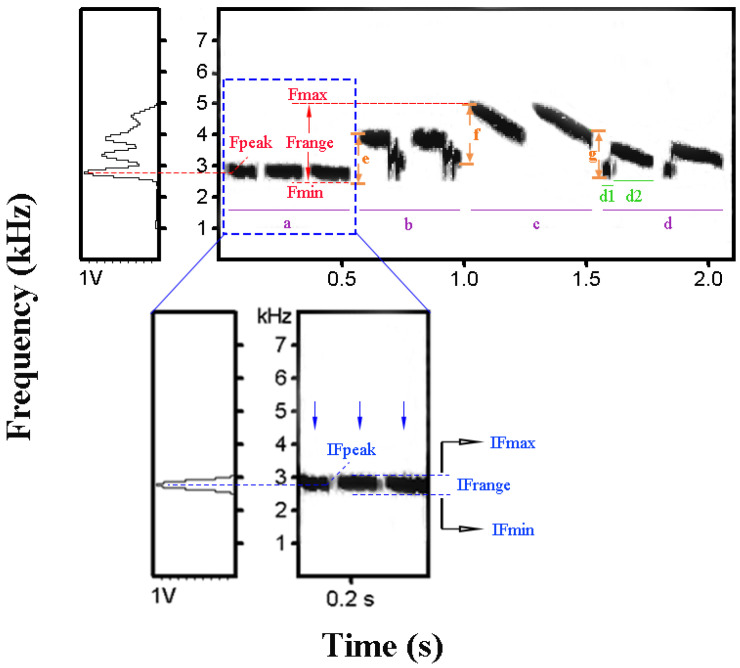
A typical song of the Yellow-breasted Bunting *Emberiza aureola* showing an obvious introductory phrase at the beginning, a repeated phrase in which the same syllable appears repeatedly and modulation frequency of adjacent phrases. “a” represents the introductory phrase. “a” “b” “c” and “d” indicate different repeated phrases. The blue arrows indicate three syllables in repeated phrase “a” “d1” and “d2” indicate different elements that are discontinuous tracks in the sonogram of a syllable. “e” “f” and “g” show frequency modulation between neighboring phrases. As for “f” MFpeak is equal to the difference in the peak frequency of “b” and “c”, which is divided by the duration from the start of “b” to the end of “c”; MFrange is equal to the difference in the frequency bandwidth of “b” and “c”, which is divided by the duration from the start of “b” to the end of “c”; and MFmaxmin is equal to the difference in frequency between the end of “b” and the start of “c”, which is divided by the interval between “b” and “c”.

In the measurement process for each individual, good quality phrases were randomly selected from different song types as much as possible. Three to five songs of high quality were measured in Avisoft-SASLab Pro 4.52 (Avisoft Bioacoustics, Berlin, Germany), and average values were taken to stand for each acoustic parameter. In Avisoft, the FFT length is 256, window type is FlatTop and overlapping level is 75%. YBB sing frequently and continuously in the breeding season at several spots, and introductory phrases (syllable combination), repeated phrases, and obvious frequency shifts between neighboring phrases are typical vocal characteristics (Figure 2). We measured 25 parameters associated with 4 traits of YBB song: complete songs, introductory phrases, repeated phrases, and frequency modulation. Introductory phrases were defined by syllable combination at the beginning of each sentence. Repeated phrases are typical traits of YBB song made up of several consecutive identical syllables that appear repeatedly in one song. Frequency modulation was defined by significant frequency changes between two adjacent phrases. All vocal parameter measurements are shown in Figure 2.

For complete songs, we measured six parameters: (1) maximum frequency (Fmax): the highest frequency in the song, (2) minimum frequency (Fmin): the lowest frequency in the song, (3) bandwidth (Frange): the difference between Fmax and Fmin, (4) peak frequency (Fpeak): the frequency at the maximum amplitude of the song, (5) duration (D): the duration of the song, and (6) Rate: the number of syllables divided by the duration of the song.

For introductory phrases, we measured eight parameters: (7) IN: the number of syllables in the introductory phrase, (8) IFmax: the highest frequency in the introductory phrase, (9) IFmin: the lowest frequency in the introductory phrase, (10) IFrange: the difference between IFmax and IFmin, (11) IFpeak: the frequency at the maximum amplitude of the introductory phrase, (12) ID: the duration of the introductory phrase, (13) IRate: the number of syllables divided by the duration of the introductory phrase (IN/ID), and (14) IDR: the duration of the introductory phrase divided by the duration of the song (ID/D).

For repeated phrases, we measured six parameters: (15) RN: the number of repeated phrases, (16) RNS: the number of syllables in each repeated phrase, (17) RNE: the average number of elements in one syllable of the repeated phrase, (18) RD: the duration of each repeated phrase, (19) RDR: the total duration of the repeated phrase divided by the duration of the song, and (20) RSRate: the number of syllables in each repeated phrase divided by the duration of the respective repeated phrase.

For frequency modulation, we measured five parameters: (21) MN: the number of frequency modulations, (22) MRate: the number of frequency modulations divided by the duration of the song (MN/D), (23) MFpeak: the difference in peak frequency of two adjacent phrases divided by their duration, (24) MFrange: the bandwidth frequency of two adjacent phrases divided by their duration, and (25) MFmaxmin: the difference in frequency of two adjacent phrases divided by the interval from the end of the first phrase to the start of the second adjacent phrase.

### 2.3. Statistical Analysis

We investigated song differences between subspecies and tested whether *insulana* distributed in Hokkaido, Japan, differed from the other two subspecies to determine the taxonomic status of *insulana*. We tested differences in song among populations to determine whether any unique population acoustics may indicate a priority for conservation. All parameters were analyzed in nine populations of three subspecies. Firstly, Kaiser–Meyer–Olkin (KMO) and Bartlett’s Test were performed on all data to test whether they were suitable for factor analysis. The results showed that for subspecies or population data, KMO values were all greater than 0.5 and *P* values were all less than 0.05 in Bartlett’s Test (Table S2). In addition, we performed principal component analysis (PCA) to reduce the number of variables to compare and the eigenvalues of the principal components are all greater than 1. Seven principal components were extracted at both subspecies (cumulative proportion 82.7%) and population (cumulative proportion 82.5%) levels (Tables S3 and S4) and generalized linear model (GLM) analysis was performed using the extracted PCA to test for differences between subspecies and populations.. Potential discrimination among songs from subspecies and populations was tested with linear discriminant analysis (LDA), which is performed by using the first three principal components. LDA is a multivariate technique that fits orthogonal, linear functions from a series of predictor variables to divide individuals into assigned categorical groups with the least amount of error. To further analyze which specific parameters differ among subspecies and populations, the Shapiro–Wilk test was used to test for normality of variables. Analysis of variance (ANOVA) was performed for parameters conforming to normal distribution, otherwise Kruskal–Wallis rank sum test was used. Statistical analyses were performed in SPSS v25 (IBM, Chicago, IL, USA) and R ver. 4.0.5 (R Foundation for Statistical Computing, Vienna, Austria, http://www.r-project.org, accessed on 2 September 2021).

## 3. Results

### 3.1. Song Divergences at the Subspecies Level

**Figure 3:**
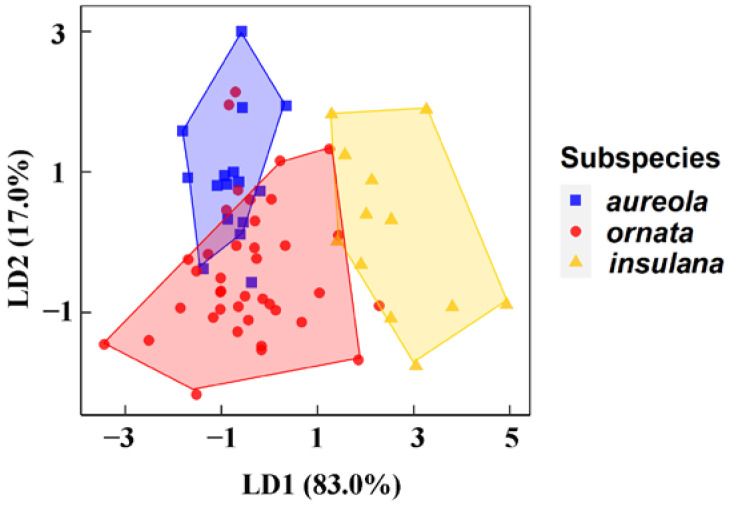
Linear discriminant analysis (LDA) indicated that the three subspecies could be separated completely by the first two discriminant functions.

According to GLM, the differences of the extracted principal component 2 between subspecies were very significant (Table 1). In PC2, Fmin, IN, IFmin, ID, RN, RNS, RNE, RDR, and MFmaxmin contribute more (Table S3). The LDA (Figure 3 and Table S7) classified the three subspecies mainly by PC2 of LD1 (explaining 83.0% of the total variance) and PC1 of LD2 (explaining 17.0% of the total variance). ANOVA and Kruskal–Wallis rank sum tests revealed that 18 of 25 parameters showed significant acoustic differences among the three subspecies (Tables S5 and S6). There are significant differences in *insulana* distributed in Japan from the other two subspecies in multiple song parameters. This was mainly reflected in shorter duration, including ID (Table S6, χ^2^ = 7.797, *p* < 0.05) and RD (Table S6, χ^2^ = 15.763, *p* < 0.001); a lower syllable rate, including Rate (Table S5, *F* [2, 86] = 33.350, *p* < 0.001), IRate (Table S6, χ^2^ = 6.672, *p* < 0.05), and RSRate (Table S6, χ^2^ = 32.519, *p* < 0.001); a higher frequency, including Fmin (Table S5, *F* [2, 86] = 11.110, *p* < 0.001), IFmax (Table S6, χ^2^ = 29.875, *p* < 0.001), IFmin (Table S6, χ^2^ = 18.889, *p* < 0.001), IFrange (Table S6, χ^2^ = 19.909, *p* < 0.001), and IFpeak (Table S6, χ^2^ = 16.114, *p* < 0.001); and less frequency modulation, including MFmaxmin (Table S5, *F* [2, 86] = 5.397, *p* < 0.01).

**Figure 4:**
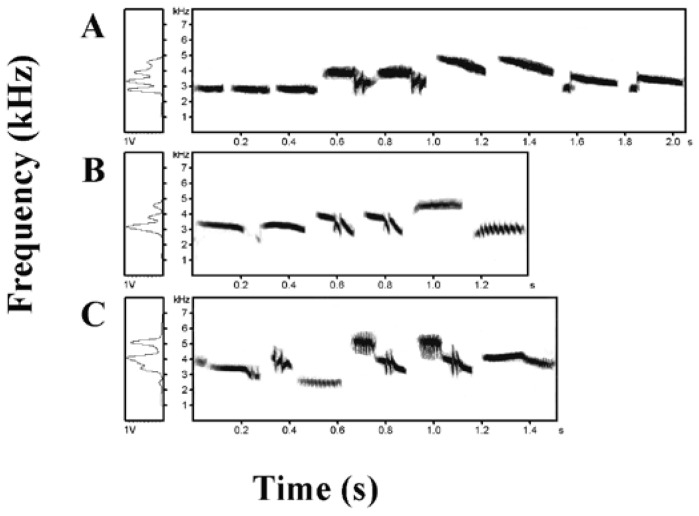
The typical song of each subspecies. (**A**) is from *E. a. aureola*, (**B**) is from *E. a. ornata* and (**C**) is from *E. a. insulana*.

Song divergences among the *insulana*, *aureola*, and *ornata* subspecies were clearly shown in all acoustic characteristics measured in the study (Figure 4). As for the whole song, the greatest divergence at the subspecies level was between *insulana* and the other two subspecies (Tables S5 and S6): *insulana* had the highest Fmin and slowest syllable delivery (Rate). As for introductory phrases, *aureola* had the longest song duration (ID), the most syllables (IN), and the slowest syllable delivery (IRate); *insulana* had the fewest syllables (IN) and the highest frequency (IFmax, IFmin, IFpeak); and *ornata* had the lowest frequency (IFmax, IFmin, IFpeak) and the narrowest frequency bandwidth (IFrange). As for repeated phrases, *insulana* was low in repeated singing style, with the fewest number and slowest rate of repeated phrases (RN and RDR), fewest syllables (RNS), and slowest syllable delivery (RSRate). However, *aureola* was high in repeated singing style, with the highest number and fastest rate of repeated phrases (RN and RDR), longest duration (RD), and most syllables (RNS). As for frequency modulation, *insulana* and *aureola* were lower in frequency modulation, whereas *ornata* was higher. In particular, *insulana* had the smallest frequency variation from highest to lowest (Fmax–Fmin) between neighboring phrases. Frequency modulation was indicated by the number of frequency modulations per second, peak frequency variation, and frequency variation from highest to lowest (Fmax–Fmin) between neighboring phrases.

### 3.2. Song Variations among Geographic Populations

**Figure 5:**
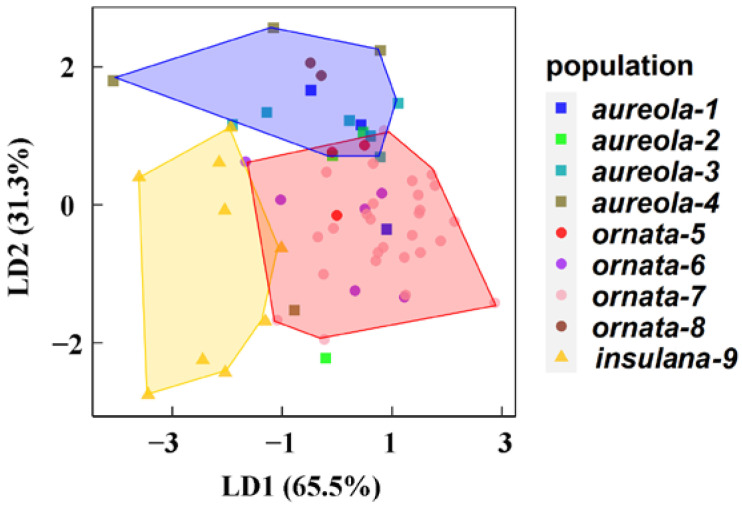
LDA indicated that the nine populations could be separated completely by the first two discriminant functions.

After we deleted populations with only one individual, nine populations of three subspecies were reanalyzed to test vocal differences at the population level. According to GLM, the differences of the extracted principal component 2 between populations were very significant (Table 2). In PC2, Fmin; IN; IFmin; ID; RN; RNS; RNE; and MFmaxmin contribute more (Table S4). Based on significant song divergences among the three subspecies, we analyzed variations in song among the different geographic populations. The results of LDA suggested that the nine populations could be clearly distinguished according to the three subspecies, and populations of *insulana* were clearly distinct from populations of *aureola* and *ornata* (Figure 5). They were mainly distinguished by PC1 of LD1 (explaining 65.5% of the total variance) and PC2 of LD2 (explaining 31.3% of the total variance; Table S10). ANOVA and Kruskal–Wallis rank sum tests revealed that 15 of 25 parameters showed significant acoustic differences among the nine populations (Tables S8 and S9).

Several unique populations were not fully clustered closely according to their subspecies status or geographic location (Figure 5). Population 1 of *aureola* (Sverdlovsk Oblast, Russia) was much closer to the far populations 5 (northeastern and eastern Mongolia), 6 (Zhalong, Heilongjiang Province, China), and 7 (Muraviovka Park, Russian Far East) of the subspecies *ornata* than populations 3 (Buryatiya, Russia) and 4 (Baikal, Russia) of the same subspecies *aureola*. Population 2 of *aureola* (Xinjiang, China) was much closer to populations 5 (northeastern and eastern Mongolia), 6 (Zhalong, Heilongjiang Province, China), and 7 (Muraviovka Park, Russian Far East) of the subspecies *ornata* than populations 3 (Buryatiya, Russia) and 4 (Baikal, Russia) of the same subspecies *aureola*.

It is worth noting that population 8 (Khasyn, Magadanskaya Oblast, Russia) of *ornata* was close to populations of *aureola*, but it could be clearly distinguished from other populations of *ornata* (Figure 5), mainly in IN, ID, IRate, RNS, RD, and MFmaxmin (Tables S8 and S9).

## 4. Discussion

Results of acoustic comparisons in this research indicate that YBB song has both significant subspecies-specific and population-specific signatures and support the claim that the subspecies *insulana* is diagnosed from the subspecies *aureola* and *ornata*.

### 4.1. Taxonomy by Subspecies Status

It is clear that YBB song has distinct subspecies-specific characteristics among *aureola*, *ornata*, and *insulana*. LDA clearly distinguishes the subspecies *aureola* and *ornata* from each other, in line with the broadly accepted taxonomy with *aureola* and *ornata* as two subspecies [8,9]. The quantitative properties of introductory phrases, repeated phrases, and frequency modulation among neighboring phrases all contributed to this pattern. These results suggest that YBB song has distinct subspecies-specific traits and demonstrate that the acoustic parameters in this research are effective at distinguishing YBB subspecies. Different subspecies may have various morphological or life history traits or may be subject to different selection pressures, which may lead to differences in song at the subspecies level that have profound consequences for segregation within a species [15,17,18]. All three subspecies have unique song signals that can distinguish them from one another. These results indicate that YBB subspecies recognition and male quality assessment may depend on multiple parameters [34].

The subspecies *aureola* and *ornata* were classified clearly by LD1 and LD2 in LDA. The subspecies *aureola* sings long introductory phrases and more repeated phrases. It is common for many songbirds to produce introductory vocalizations before the start of complex songs to increase detectability, such as in noisy backgrounds, or provide signals for recognition, such as local-dialect identity [35,36]. The addition of introductory vocalizations before the song increases the magnitude of the male Rufous-sided Towhee’s response [35]. The function of the introductory phrase of the *aureola* song and how it contributes to subspecies divergence under selection needs further examination in song playback experiments. In terms of repeated phrases, urban Song Thrush repeat syllables more often than conspecifics from natural forest populations, and this may be an adaptation of acoustic communication in noisy urban environments [37]. Using evidence from common features of both introductory phrases and repeated phrases, researchers can test whether the subspecies *aureola* inhabits a noisier environment, compared to other subspecies.

Songs of the subspecies *ornata* are of the shortest duration, have a low frequency, and have a narrow bandwidth of introductory phrase and repeated phrase but the most frequency modulation. It is interesting that the low frequency of *ornata*’s introductory phrase goes against the classic acoustic adaptation hypothesis [24,38,39]. Songs of low frequency are expected to transmit better through dense vegetation, but *ornata* inhabits areas with less shrub cover (40–55%), compared to *aureola* (50–90%) [4]. There is another possibility to explain this nonconformity: the open canopy shrub cover probably leads to greater distance between each territory of *ornata* and lower frequency is advantageous to broadcasting farther. Frequency modulation has been found in other bird species, and several lines of evidence focus on the potential role of frequency modulation as an assessment signal [40,41]. For example, it can evoke an increased response in Eurasian Collared Dove [34]. If frequency modulation reflects the accurate assessment for *ornata* male, why *ornata* uses frequency modulation to signal its competitive potential is still unclear.

After correct and exact classification of *aureola* and *ornata*, we used the same multiple acoustic parameters to test the taxonomic status of *insulana*. The results of LDA showed that *insulana* distributed in Hokkaido, Japan, was clearly separate from *aureola* and *ornata*, which indicates its independent status as a third subspecies vocally. Island populations of other bird species are known to have unique song dialects, compared to mainland populations [42,43]. In this study, *insulana* distributed in Japan diverged significantly from *aureola* and *ornata* on multiple song parameters: it had shorter durations of introductory (ID) and repeated (RD) phrases, lower syllable delivery rates, higher frequency, and less frequency modulation. These acoustic divergences may be caused by multiple factors. First, there is evidence that YBB breeding in Japan overlap or have the opportunity to meet with *ornata* distributed in China and the Russian Far East during migration [44]. However, the vocal differences between *insulana* and *ornata* in this study indicate no song learning from each other and again may support the independence of the local *insulana*. The acoustic adaptation hypothesis can better explain the distinct song characteristics of *insulana*. Populations of *insulana* are distributed in Hokkaido, northern Japan, an island, which, typically have stronger winds. The higher frequency of their song may serve to prevent them from being masked like other birds, such as skylark, that live in windy environments [45]. High-frequency songs can also be found among urban birds singing at a higher pitch to avoid being masked by low-frequency traffic noise [46]. Second, there is evidence that birds of island populations sing much simpler songs than mainland birds [47], and the lower syllable delivery rates and low frequency modulation found among *insulana* are in line with these findings [48,49]. However, whether the distinct song of *insulana* is a premating isolation barrier or whether it can be recognized by other subspecies is still unknown and must be tested in song playback studies. Morphological effects on vocal differences among subspecies should also be studied in the future [50].

Given that all three subspecies of YBB have vocal properties that differentiate them from one another, future researchers should consider how these variations in song promote divergences within this endangered species faced with population collapse. Song traits are likely to be targets of selection, which potentially lead to an evolutionary response and sexual selection. Song divergence promoting the evolution of reproductive isolation has been found in many bird species [51,52]. Especially when there is greater variation in song between subspecies related to female attraction, there is a greater potential for premating reproductive isolation to evolve [15,18,53,54].

### 4.2. Song Variations among Geographic Populations

LDA (Figure 5) showed that all nine populations of three subspecies maintained clear population level boundaries and demonstrated that YBB song has a population-specific signature. These results support the significant vocal divergences among *aureola*, *ornata*, and *insulana* and support the status of *insulana* as a subspecies independent from *aureola* and *ornata*. Given the acoustic independence of *insulana*, populations in Japan should be paid more attention in terms of conservation, because several populations in Hokkaido, Japan, have completely disappeared since the 1990s, which is a severe problem [55,56].

Birdsongs are important for sexual selection and species recognition among birds [12]. Geographic variations among populations can allow individuals to distinguish local neighbors from immigrants or intruders to avoid mating with maladapted individuals [57,58]. Several mechanisms have been proposed to explain how birds maintain their population-specific song dialects and how a low level of dispersal and strong assortative mating ensures that dialects are maintained [59]. The successful recovery of YBB with geolocators and color-rings in Mongolia and Muraviovka Park, Russia indicated that their breeding site fidelity may lead to stable song dialectal patterns at the population level [60,61].

However, it is worth noting that a few populations of *aureola* and *ornata* were not close according to their subspecies taxonomic status. Populations 1 and 2 of *aureola* were not clustered with populations 3 or 4 of the same subspecies but rather clustered with populations 5, 6, and 7 of the subspecies *ornata*. It may be the case that population 1 had too few individuals and thus clustered closer to the geographically farther *ornata* population; more individual local songs should be included in further analyses. As for population 2, it clustered first with populations 5, 6, and 7 of the subspecies *ornata*, not with the geographically closer populations 3 and 4 of the same subspecies, which may indicate unique vocal differentiation within *aureola*. The small number of recordings from populations 1 and 2 might have led to findings of song variations within *aureola*; more songs should be collected from these populations for future in-depth studies. However, the results of these few individuals from the same population clustered together may indicate the unique life history or local vocal adaptation of populations 1 and 2. Further study is needed to investigate the formation mechanisms to make timely conservation efforts to save these special local taxa.

Studies of dialects and other characteristics of song can reveal important information about the ecology of bird species because geographic song variations are often regarded as a potential first step in speciation [32]. For example, the intensity of song variants or syllable sharing can indicate gene flow or a degree of isolation among geographic populations [62,63]. A loss of song variability may reflect population decline [1,64,65]. Therefore, it is essential to continuously monitor variations in song and sharing patterns among geographic populations of YBB, and populations distributed in Kamchatka should also be included in future study.

## 5. Conclusions

The acoustic analysis in this research clearly supports the existence of three subspecies in YBB: it not only distinguishes *aureola* from *ornata* but also supports the song divergence of *insulana* distributed in Japan from *aureola* and *ornata*. Genetic analyses and other phenotypic analyses are essential to confirm the subspecies status of *insulana* and the genetic diversity of the critically endangered YBB in the future. YBB song has both subspecies-specific and population-specific signatures, which indicates that conserving YBB globally can also conserve the vocal cultural diversity of this critically endangered bird species. Because the YBB population is on the verge of collapse, all subspecies and populations need to be conserved, but some unique populations may be given priority. For conservation practice globally, studies of life history, and migration, and monitoring of wintering birds are urgently needed to avoid local extinction or dramatic decreases in population.

## Figures and Tables

**Table 1:** GLM results obtained by extracting principal components for all song parameters of subspecies.

Variable	Estimate	*SE*	*t*	*p*
Intercept	1.944	0.059	33.049	0.000 ***
Principal component 1	0.018	0.059	0.311	0.757
Principal component 2	−0.336	0.059	−5.678	0.000 ***
Principal component 3	−0.052	0.059	-0.887	0.378
Principal component 4	−0.070	0.059	−1.187	0.239
Principal component 5	−0.006	0.059	−0.094	0.925
Principal component 6	−0.113	0.059	−1.909	0.060
Principal component 7	−0.017	0.059	−0.292	0.771

*** *p* < 0.001.

**Table 2:** GLM results obtained by extracting principal components the all song parameters of populations.

Variable	Estimate	*SE*	*t*	*p*
Intercept	6.088	0.225	27.028	0.000 ***
Principal component 1	−0.186	0.227	−0.823	0.413
Principal component 2	−0.933	0.227	−4.115	0.000 ***
Principal component 3	−0.257	0.227	−1.134	0.261
Principal component 4	−0.326	0.227	−1.440	0.154
Principal component 5	0.103	0.227	0.456	0.650
Principal component 6	−0.280	0.227	−1.237	0.220
Principal component 7	0.018	0.227	0.078	0.938

*** *p* < 0.001.

## Data Availability

The data presented in this study are available on request from the corresponding author. The data are not publicly available due to privacy and ethical restrictions.

## Supplementary Materials

The following supporting information can be downloaded at: https://www.mdpi.com/article/10.3390/ani12172292/s1, Table S1: The information of all subspecies, population location and individual numbers of song recordings. Songs used in population divergence analysis are indicated in bold; Table S2: KMO and Bartlett’s test results among subspecies and population; Table S3: Loadings and cumulative proportions of principal components of each parameter among subspecies; Table S4: Loadings and cumulative proportions of principal components of each parameter among populations; Table S5: Means of acoustic parameters of song which are shown ± *SD* and results of one-way ANOVA showing significant differences among 3 subspecies. N is the number of individuals; Table S6: Means of acoustic parameters of song which are shown ± SD and results of Kruskal-Wallis rank sum test showing significant differences among 3 subspecies. N is the number of individuals; Table S7: Linear discriminant analysis (LDA) of three subspecies. Percent variance, and the coefficients of linear discriminants of principal components; Table S8: Means of acoustic parameters of song which are shown ± *SD* and results of one-way ANOVA showing significant differences among 9 populations. N is the number of individuals; Table S9: Means of acoustic parameters of song which are shown ± *SD* and results of Kruskal-Wallis rank sum test showing significant differences among 9 populations. N is the number of individuals; Table S10: Linear discriminant analysis (LDA) of nine populations. Percent variance, and the coefficients of linear discriminants of principal components.
