# Supplementary material for: Subspecies Taxonomy and Inter-Population Divergences of the Critically Endangered Yellow-Breasted Bunting: Evidence from Song Variations
Source: Animals (Basel). 2022 Sep 4;12(17):2292. doi: 10.3390/ani12172292 (PMC9454650; doi:10.3390/ani12172292)
Supplement: Supplementary file 1 [file animals-12-02292-s001.zip › animals-1866195-supplementary-updated.pdf]

Table S1. The information of all subspecies, population location and individual numbers of song recordings. Songs used in population divergence analysis are indicated in bold.

Subspecies	Populations	Location	Number of individuals
E. a. aureola	**1**	**Sverdlovsk Oblast, Russia**	**3**
	**2**	**Qinghe County, Xinjiang province, China**	**4**
	**3**	**Buryatiya, Russia**	**8**
	**4**	**Lake Baikal, Russia**	**5**
	5	Northern Ostrobothnia, Finland	1
E. a. ornata	**6**	**Northeastern and eastern Mongolia**	**3**
	**7**	**Zhalong, Heilongjiang Province, China**	**8**
	8	Xingkai Lake, Heilongjiang Province, China	1
	9	Amurskaya Oblast, Russia	1
	**10**	**Muraviovka Park, The Russian Far East**	**35**
	11	Khabarovsk, Russia	1
	**12**	**Khasyn, Magadanskaya Oblast, Russia**	**2**
	13	Lena Valley Sakha, Respublika, Russia	1
E. a. insulana	14	Mombetsu, Hokkaido, Japan	1
	15	Toyokoro, Hokkaido, Japan	1
	16	Ikeda, Hokkaido, Japan	1
	**17**	**Toyotomi, Hokkaido, Japan**	**12**
	18	Yubetsu, Hokkaido, Japan	1

Table S2. KMO and Bartlett's test results among subspecies and population.

	**Kaiser-Meyer-Olkin**	**Bartlett's Test**		
	F	χ^2	df	*P*
Subspecies	0.587	4789.250	300	0.000
Population	0.601	4341.474	300	0.000

Table S3. Loadings and cumulative proportions of principal components of each parameter among subspecies.

**Variables**	**PC1 (28.2%)**	**PC2 (16.9%)**	**PC3 (12.5%)**	**PC4 (9.0)**	**PC5 (6.1%)**	**PC6 (5.2%)**	**PC7 (4.7%)**
Cumulative proportion	28.2%	45.1%	57.6%	66.6%	72.7%	77.9%	82.7%

Eigenvalue	7.047	4.229	3.114	2.262	1.534	1.301	1.179
Fmax	-0.022	-0.021	0.237	-0.093	0.193	0.204	0.218
Fmin	0.091	-0.122	-0.027	0.162	0.007	0.091	0.005
Frange	-0.062	0.039	0.217	-0.156	0.162	0.133	0.185
Fpeak	0.005	-0.092	-0.071	0.015	0.210	0.317	0.296
D	-0.051	0.069	0.101	-0.052	-0.310	0.436	-0.238
Rate	-0.081	0.079	-0.048	0.286	0.120	-0.020	0.076
IN	0.038	0.182	0.059	0.155	-0.112	0.032	0.161
IFmax	0.098	-0.088	0.126	0.137	0.116	0.108	-0.171
IFmin	0.081	-0.141	-0.001	0.168	-0.034	0.137	0.005
IFrange	0.072	-0.009	0.178	0.056	0.191	0.041	-0.245
IFpeak	0.095	-0.080	0.081	0.179	0.104	0.157	-0.122
ID	0.089	0.144	0.104	0.014	-0.123	-0.040	-0.022
IRate	-0.084	-0.045	-0.099	0.110	0.045	0.161	0.270
IDR	0.103	0.099	0.051	0.083	0.018	-0.266	-0.007
RN	-0.056	0.139	-0.094	0.033	0.154	0.212	-0.322
RNS	0.056	0.130	0.010	0.173	-0.196	0.180	0.325
RNE	0.054	-0.108	-0.022	-0.137	-0.115	-0.043	0.324
RD	0.101	0.076	0.065	-0.082	-0.205	0.095	0.176
RDR	0.023	0.133	-0.117	-0.044	0.295	0.125	-0.088
RSRate	-0.087	0.059	-0.038	0.271	0.011	0.011	0.115
MN	-0.111	-0.041	0.118	0.040	-0.207	0.147	-0.126
MRate	-0.113	-0.091	0.091	0.079	-0.087	-0.049	-0.023
MFpeak	-0.083	-0.047	0.165	0.089	-0.038	-0.167	-0.033
MFrange	-0.052	-0.031	0.150	0.151	-0.010	-0.297	0.057
MFmaxmin	-0.016	0.112	0.135	-0.042	0.291	-0.101	0.163

Table S4. Loadings and cumulative proportions of principal components of each parameter among populations.

**Variables**	**PC1 (27.8%)**	**PC2 (17.2%)**	**PC3 (12.6%)**	**PC4 (8.7%)**	**PC5 (6.4%)**	**PC6 (5.4%)**	**PC7 (4.5%)**
Cumulative proportion	27.8%	45.0%	57.6%	66.2%	72.6%	78.0%	82.5%
Eigenvalue	6.952	4.301	3.139	2.163	1.599	1.355	1.113
Fmax	-0.021	-0.013	0.242	-0.037	0.143	0.238	0.256

Fmin	0.086	-0.135	-0.038	0.164	0.004	0.076	-0.004
Frange	-0.058	0.052	0.227	-0.109	0.122	0.171	0.224
Fpeak	0.004	-0.104	-0.080	-0.029	0.146	0.351	0.207
D	-0.046	0.086	0.089	-0.006	-0.351	0.363	-0.261
Rate	-0.076	0.070	-0.069	0.316	0.129	-0.032	0.103
IN	0.057	0.171	0.025	0.151	-0.118	0.010	0.162
IFmax	0.101	-0.089	0.112	0.145	0.104	0.130	-0.155
IFmin	0.079	-0.147	-0.017	0.175	-0.054	0.096	0.013
IFrange	0.079	-0.009	0.170	0.064	0.186	0.106	-0.226
IFpeak	0.098	-0.086	0.064	0.188	0.077	0.143	-0.123
ID	0.097	0.137	0.084	0.019	-0.108	-0.052	-0.047
IRate	-0.081	-0.054	-0.107	0.080	0.008	0.157	0.323
IDR	0.109	0.089	0.031	0.079	0.061	-0.257	-0.007
RN	-0.055	0.140	-0.089	0.055	0.139	0.217	-0.344
RNS	0.070	0.109	-0.019	0.176	-0.224	0.117	0.343
RNE	0.045	-0.123	-0.012	-0.145	-0.124	-0.050	0.255
RD	0.105	0.072	0.061	-0.064	-0.217	0.068	0.180
RDR	0.022	0.123	-0.105	-0.052	0.282	0.164	-0.085
RSRate	-0.080	0.055	-0.084	0.285	0.026	0.023	0.142
MN	-0.111	-0.023	0.116	0.081	-0.227	0.114	-0.139
MRate	-0.116	-0.079	0.092	0.105	-0.080	-0.051	-0.018
MFpeak	-0.076	-0.049	0.176	0.085	-0.025	-0.168	-0.074
MFrange	-0.044	-0.034	0.144	0.160	0.030	-0.308	0.019
MFmaxmin	-0.008	0.109	0.137	-0.027	0.271	-0.071	0.226

Table S5. Means of acoustic parameters of song which are shown ± *SD* and results of one-way ANOVA showing significant differences among 3 subspecies. N is the number of individuals.

	Fmax	Fmin	Frange	D	Rate	RN	MFmaxmin
aureola (N=21)	5624±340	1957±288	3668±466	1.8±0.2	4.1±0.4	2.6±0.5	41453±9371
ornata (N=52)	5604±541	1940±230	3664±595	1.7±0.3	4.4±0.4	2.7±0.7	39357±9127
insulana (N=16)	5646±514	2258±205	3388±592	1.7±0.3	3.5±0.2	1.8±0.6	31736±10294
**ANOVA**							
$F_{(2,86)}$	0.047	11.110	1.565	2.502	33.350	12.630	5.397
P	0.954	0.000	0.215	0.088	0.000	0.000	0.006

Table S6. Means of acoustic parameters of song which are shown ± SD and results of Kruskal-Wallis rank sum test showing significant differences among 3 subspecies. N is the number of individuals.

	Fpeak	IN	IFmax	IFmin	IFrange	IFpeak	ID	IRate	IDR	RNS	RNE	RD	RDR	RSRate	MN	Mrate	MFpeak	MFrange
aureola (N=21)	3580±566	2.4±0.5	3390±775	2100±448	1290±543	2861±761	0.6±0.2	4.5±0.8	0.3±0.1	2.4±0.4	1.9±0.4	0.5±0.1	0.7±0.1	4.5±0.4	3.2±0.9	1.7±0.5	1154±439	1071±376
ornata (N=52)	3584±471	2.1±0.5	2882±447	2031±288	850±304	2468±349	0.4±0.1	5.0±1.0	0.3±0.1	2.2±0.3	1.7±0.3	0.4±0.1	0.7±0.2	5.2±0.9	3.6±1.1	2.1±0.5	1558±568	1269±479
insulana (N=16)	3870±544	1.6±0.6	3715±464	2458±290	1256±473	2939±396	0.4±0.2	4.6±1.7	0.3±0.1	2.0±0.2	2.2±0.5	0.5±0.1	0.5±0.2	3.9±0.6	3.0±0.9	1.8±0.3	1370±333	1100±428
**Kruskal-Wallis rank sum test**																		
χ^2	4.232	17.302	29.875	18.889	19.909	16.114	7.797	6.672	2.290	23.226	14.023	15.763	10.425	32.519	5.163	8.237	8.685	2.783
p	0.121	0.000	0.000	0.000	0.000	0.000	0.020	0.036	0.318	0.000	0.001	0.000	0.005	0.000	0.076	0.016	0.013	0.249

Table S7. Linear discriminant analysis (LDA) of three subspecies. Percent variance, and the coefficients of linear discriminants of principal components.

	Percent variance (%)	PC1	PC2	PC3
LD1	83.0	0.738663	-1.37218	-0.0257
LD2	17.0	0.943009	0.3857	0.571399

Table S8. Means of acoustic parameters of song which are shown ± *SD* and results of one-way ANOVA showing significant differences among 9 populations. N is the number of individuals.

	Fmax	Frange	D	Rate	RN	RSRate	MFmaxmin
1 (N=3)	5386±158	3349±236	1.9±0.1	4.2±0.3	2.7±0.4	4.9±0.2	39102±7925
2 (N=4)	5404±261	3517±315	1.8±0.1	4.0±0.2	2.4±0.4	4.6±0.4	40198±9656
3 (N=8)	5795±355	3934±461	1.9±0.2	4.2±0.4	2.7±0.5	4.6±0.4	44903±7218
4 (N=5)	5686±374	3547±594	1.8±0.2	4.1±0.4	2.5±0.6	4.4±0.6	38655±14377
5 (N=3)	5430±107	3701±39	1.8±0.3	4.0±0.4	2.3±0.3	4.2±0.5	43754±4932

6 (N=8)	5597±495	3522±318	1.7±0.2	4.6±0.3	2.9±0.8	5.3±0.5	39343±7593
7 (N=35)	5575±584	3663±675	1.8±0.3	4.4±0.4	2.8±0.7	5.2±0.6	37369±8806
8 (N=2)	5870±113	3741±77	1.8±0.0	3.9±0.2	2.3±0.1	4.6±0.4	57757±601
9 (N=12)	5648±577	3343±632	1.6±0.3	3.5±0.2	1.7±0.6	3.9±0.6	30552±10035
**ANOVA**							
$F_{(8,71)}$	0.433	0.805	1.048	10.320	3.486	7.602	3.020
P	0.898	0.600	0.409	0.000	0.002	0.000	0.006

Table S9. Means of acoustic parameters of song which are shown ± *SD* and results of Kruskal-Wallis rank sum test showing significant differences among 9 populations. N is the number of individuals.

	Fmin	Fpeak	IN	IFmax	IFmin	IFrange	IFpeak	ID	IRate	IDR	RNS	RNE	RD	RDR	MN	Mrate	MFpeak	MFrange
1 (N=3)	2037±173	3269±287	2.7±0.6	3369±389	2103±242	1266±287	2883±370	0.6±0.1	4.8±0.6	0.3±0.1	2.4±0.5	1.6±0.0	0.5±0.2	0.7±0.1	3.4±1.1	1.8±0.6	1057±266	1180±71
2 (N=4)	1887±68	3791±804	2.0±0.7	3034±374	1968±35	1067±385	2400±206	0.5±0.2	5.0±1.2	0.3±0.1	2.2±0.2	2.1±0.3	0.5±0.1	0.7±0.1	3.2±0.2	1.8±0.1	1124±388	1029±188
3 (N=8)	1861±277	3473±591	2.3±0.3	3305±859	2038±397	1267±742	2825±878	0.6±0.1	4.2±0.3	0.3±0.1	2.3±0.4	1.8±0.3	0.5±0.1	0.7±0.1	3.6±1.0	1.9±0.5	1251±464	1277±308
4 (N=5)	2138±438	3882±362	2.7±0.5	3892±1019	2353±760	1539±474	3368±925	0.6±0.2	4.5±1.0	0.4±0.1	2.6±0.4	1.8±0.5	0.6±0.2	0.8±0.1	2.7±1.2	1.5±0.5	1172±598	808±516
5 (N=3)	1729±145	3158±389	1.9±0.1	3107±149	2007±131	1101±192	2667±226	0.5±0.1	3.9±0.6	0.3±0.0	2.0±0.0	1.8±0.3	0.5±0.1	0.6±0.1	3.7±0.8	2.1±0.1	2187±386	1541±527
6 (N=8)	2075±215	3877±593	2.2±0.4	3276±662	2152±245	1124±524	2654±361	0.4±0.1	5.4±1.0	0.3±0.1	2.1±0.1	1.7±0.3	0.4±0.1	0.7±0.1	3.4±0.5	2.0±0.2	1630±540	1296±542
7 (N=35)	1913±218	3538±446	2.0±0.5	2721±328	1991±298	729±177	2380±342	0.4±0.1	5.1±0.8	0.3±0.1	2.1±0.3	1.7±0.3	0.4±0.1	0.7±0.3	3.7±1.3	2.1±0.5	1496±480	1184±421
8 (N=2)	2130±36	3644±479	2.9±0.1	3299±41	2130±36	1170±5.0	2784±54	0.8±0.0	3.8±0.1	0.4±0.0	2.6±0.1	1.9±0.1	0.6±0.0	0.7±0.0	2.6±0.1	1.4±0.0	1181±298	1182±289
9 (N=12)	2305±182	3965±575	1.5±0.5	3783±462	2508±265	1275±537	2991±425	0.4±0.2	4.6±2.0	0.3±0.1	1.9±0.2	2.4±0.5	0.5±0.1	0.5±0.2	2.9±0.8	1.8±0.4	1339±370	1055±485
**Kruskal-Wallis rank sum test**																		
χ^2	28.012	14.347	33.344	40.743	23.021	31.827	28.610	21.027	17.477	12.059	33.285	23.771	25.054	11.898	9.915	13.138	13.422	7.168
p	0.000	0.073	0.000	0.000	0.003	0.000	0.000	0.007	0.026	0.149	0.000	0.003	0.002	0.156	0.271	0.107	0.098	0.519

Table S10. Linear discriminant analysis (LDA) of nine populations. Percent variance, and the coefficients of linear discriminants of principal components.

	Percent variance (%)	PC1	PC2	PC3
LD1	0.6549	-1.12206	0.952843	-0.30255
LD2	0.3134	0.557806	0.837894	0.529933
LD3	0.0317	-0.39953	-0.2045	0.861926
